# Below the surface: The inner lives of TLR4 and TLR9

**DOI:** 10.1002/JLB.3MIR1218-483RR

**Published:** 2019-03-22

**Authors:** Laura Marongiu, Laura Gornati, Irene Artuso, Ivan Zanoni, Francesca Granucci

**Affiliations:** ^1^ Department of Biotechnology and Biosciences University of Milano‐Bicocca Milan Italy; ^2^ Harvard Medical School and Division of Gastroenterology Boston Children's Hospital Boston Massachusetts USA

**Keywords:** innate immunity, autoimmunity, intracellular signaling, receptors trafficking, inflammation

## Abstract

TLRs are a class of pattern recognition receptors (PRRs) that detect invading microbes by recognizing pathogen‐associated molecular patterns (PAMPs). Upon PAMP engagement, TLRs activate a signaling cascade that leads to the production of inflammatory mediators. The localization of TLRs, either on the plasma membrane or in the endolysosomal compartment, has been considered to be a fundamental aspect to determine to which ligands the receptors bind, and which transduction pathways are induced. However, new observations have challenged this view by identifying complex trafficking events that occur upon TLR‐ligand binding. These findings have highlighted the central role that endocytosis and receptor trafficking play in the regulation of the innate immune response. Here, we review the TLR4 and TLR9 transduction pathways and the importance of their different subcellular localization during the inflammatory response. Finally, we discuss the implications of TLR9 subcellular localization in autoimmunity.

## Graphical abstract

Review on the importance of TLR4 and TLR9 dynamic subcellular localization in the inflammatory responses.

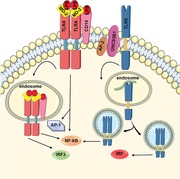

**Abbreviations**
ALRs: AIM2‐like receptors
AP‐1: activator protein 1
BAD‐LAMP: brain and DC‐associated LAMP‐like molecule
BLOC: biogenesis of lysosome‐related organelles complex
BMDC: bone marrow‐derived dendritic cells
BMDM: bone marrow‐derived macrophages
cDC: conventional dendritic cells
CIA: collagen‐induced arthritis
CLRs: C‐type lectin receptors
COPII: coat‐protein complex II
DAP12: DNAX‐activating protein of 12 kDa
DCs: dendritic cells
ER: endoplasmic reticulum
FHOD1: forming‐homology‐domain‐containing protein 1
GPI: glycosylphosphatidylinositol
HMGB: high‐mobility group box
HPS: Hermansky‐Pudlack syndrome
IKK: IκB kinase
IP3Rs: inositol trisphosphate receptors
IRAKs: IL‐1 receptor‐associated kinases
IRAP: insulin‐responsive aminopeptidase
IRF: IFN‐regulatory factor
LAMPs: lysosome‐associated membrane proteins
LPB: LPS‐binding protein
LROs: lysosome‐related organelles
MD‐2: myeloid‐differentiation protein 2
MS: multiple sclerosis
NLRs: NOD‐like receptors
OPN: osteopontin
PAMPs: pathogen‐associated molecular patterns
PBMCs: peripheral blood mononuclear cells
pDCs: plasmacytoid DCs
PI3K: phosphoinositol 3‐OH kinase
PIKfive: phosphoinositide 3‐phosphate 5‐kinase
PIs: phosphoinositides
PPRs: pattern‐recognition receptors
PRAT4A: protein associated with TLR4
RA: rheumatoid arthritis
RIG: retinoic acid‐inducible gene
RIPK1: receptor‐interacting serine/threonine kinase 1
RLRs: RIG‐I‐like receptors
Slc15a4: solute‐carrier protein superfamily member
SLE: systemic lupus erythematosus
SS: Sjögren's syndrome
SSc: systemic sclerosis
Syk: spleen tyrosine kinase
TAB: TAK‐binding protein
TAK: TGF‐β‐activated kinase
TANK: TRAF family member‐associated NF‐κB activator
TBK: TANK‐binding kinase‐1
TIR: Toll/IL‐1 receptor
TIRAP: TIR‐containing adaptor protein
TRADD: TNF receptor‐1‐associated death‐domain protein
TRAF: TNF receptor‐associated factors
TRAM: TRIF‐related adaptor molecule
TRIF: TIR‐domain‐containing adaptor inducing IFN‐β
TRPM7: transient receptor potential cation channel subfamily M member 7
UNC93B1: unc‐93 homolog B1
UPEC: uropathogenic *Escherichia Coli*
VAMPs: vesicle‐associated membrane proteins

## 1 INTRODUCTION

The innate immune system uses pattern‐recognition receptors (PRRs) to sense the presence of invading microbes. PRRs recognize endogenous and exogenous ligands, including pathogen‐associated molecular patterns (PAMPs), which are conserved chemical motifs expressed by microorganisms. According to the model proposed by Janeway, the recognition of PAMPs by PRRs is the primary strategy for self‐ versus nonself‐discrimination.^1^ Antigen‐presenting cells express high levels of PRRs that, upon ligand binding, transduce an intracellular signal, leading to the production of several factors involved in the initiation of the immune response. Ultimately, these events induce the activation of adaptive immunity and the formation of memory cells.^2^

Dysregulation of PRR‐mediated responses may compromise immunologic self‐tolerance. For example, aberrant activation triggered by host‐derived nucleic acids causes autoimmune disorders, such as Sjögren's syndrome (SS),^3^ systemic lupus erythematosus (SLE),^4^ multiple sclerosis (MS),^5^ systemic sclerosis (SSc),^6^ rheumatoid arthritis (RA),^7^ and psoriasis.^8^

PRRs are classified into 5 families: TLRs, C‐type lectin receptors (CTLs), NOD‐like receptors (NLRs), retinoic acid‐inducible gene (RIG)‐I‐like receptors (RLRs), and AIM2‐like receptors (ALRs). TLRs are the best‐characterized PPRs and are essential modulators of the innate immune response, as they survey both the intracellular and extracellular space.^9^ The widespread cellular localization of TLRs confirms their central role in recognizing potential threats and, indeed, some receptors are able to initiate signaling cascades from either the plasma membrane or endosomes.

Here, we provide an overview of the transduction pathways triggered by intracellular TLRs, with a particular focus on the signaling cascades elicited by TLR9 and the intracellular pathways of TLR4. We also discuss how TLR9 signaling may be involved in autoimmunity.

## 2 INTRACELLULAR TLRS

TLRs are glycoproteins that consist of 3 domains: a transmembrane domain, an amino‐terminal ectodomain, and a cytoplasmic carboxy‐terminal Toll IL1‐1R homology (TIR) domain.^10^, ^11^ To activate downstream signaling pathways, TLRs recruit a variety of adaptor proteins, including the TIR‐containing adaptor protein (TIRAP), MyD88, the TIR domain‐containing adaptor inducing IFN‐β (TRIF), and the TRIF‐related adaptor molecule (TRAM).^12^ Intracellular TLRs (TLR3, TLR7, TLR8, TLR9, TLR11, TLR12, and TLR13) are expressed in the endoplasmic reticulum (ER), endosomes, multivesicular bodies, and lysosomes; their localization to endosomes and lysosomes, where self‐DNA is rarely present, is important to prevent autoimmunity and inappropriate immune responses. Intracellular TLRs recognize either nucleic acids (TLR3, TLR7, TLR8, TLR9, and TLR13) or microbial components (TLR11‐TLR12), both derived from the hydrolytic degradation of microorganisms in the endolysosomal compartment.^13^

The ligand of TLR3 is double‐stranded RNA, such as that of HSV‐1, which causes encephalitis,^14^ small interfering RNAs,^15^ and self RNAs from damaged cells (e.g., RNA damaged by ultraviolet B irradiation).^16^ Similarly, TLR7 in plasmacytoid dendritic cells (pDCs) recognizes viral single‐stranded RNA, whereas it binds to the RNA of streptococcus B bacteria in conventional dendritic cells (cDCs).^17^ In addition, human TLR8 recognizes viral and bacterial RNA and is preferentially activated by ssRNA rich in AU.^18^, ^19^ On the other hand, TLR9 primarily binds unmethylated CpG DNA motifs, which are common in bacterial and viral DNA; it can also recognize hemozoin, an iron‐porphyrin‐proteinoid complex derived from the degradation of hemoglobin by malaria parasites.^20^ Parroche et al., however, proposed that hemozoin is itself immunologically inert and that its inflammatory activity is due to the presence of parasite DNA in the hemozoin crystal.^21^ Another nucleic acid‐sensing TLR, TLR13, senses bacterial 23S rRNA^22^ and vesicular stomatitis virus.^23^

Among the TLRs that recognize microbial components, TLR11 binds to an unknown proteinaceous component of uropathogenic *Escherichia coli* (UPEC)^24^ and a profilin‐like molecule derived from *Toxoplasma gondii*.^25^ TLR12 shares many similarities with TLR11: they both recognize *Toxoplasma gondii*, can form homo‐ and heterodimers, and can cooperate to recognize their ligands in cDCs and macrophages.^26^

Upon ligand binding, intracellular TLRs initiate various signaling pathways. TLR3 induces the expression of inflammatory cytokines and type I IFNs by activating TRIF‐dependent signaling through a high‐affinity interaction between its TIR domain and the TRIF domain. Notably, this binding is completely TRAM independent.^27^ On the other hand, TLR7 and TLR9 activate the transcription factor IRF7 through the MyD88‐dependent signaling pathway.^28^, ^29^ TLR3, 7, and 9 become active and trigger downstream signaling following internalization of their ectodomains into endosomes, where they undergo proteolytic cleavage. This process requires endosomal proteases and is an additional regulatory mechanism that avoids recognition of self‐molecules by strengthening the compartmentalization of intracellular TLRs.

The trafficking of intracellular TLRs from the ER to endolysosomes must be strictly controlled to ensure correct signaling cascades. Indeed, intracellular TLRs require the multimembrane protein unc‐93 homolog B1 (UNC93B1) to exit the ER and enter the secretory pathway.^30^ UNC93B1 controls the packaging of TLRs into coat protein complex II (COPII) vesicles, which then shuttle the TLRs from the ER to the Golgi.^31^ The role that UNC93B1 plays in the trafficking of TLRs is different for each receptor.^32^ Several chaperone proteins, such as glycoprotein 96 and the protein associated with TLR4 A (PRAT4A), also interact with TLRs and are important for shuttling from the ER.^33^, ^34^ Notably, nucleic acids may enter the cell through different types of endosomes and the specific site of signaling defines the final outcome of the pathway.

## 3 TLR4

Among all PRRs, TLR4 is the best characterized, as it was the first to be discovered in mammalian innate immune cells.^35^ Despite TLR4 mainly residing in the plasma membrane, it can also be considered as an intracellular TLR, because it can be internalized and stimulate intracellular pathways.^36^ Moreover, although still controversial, it has been proposed that TLR2 also activates NF‐kB from endosomes in human monocytes^37^ and induces the production of type I IFN in mouse Ly6C^high^ inflammatory monocytes in response to viral ligands.^38^ Thus, the endocytic machinery assumes a pivotal role in the regulation of pathways elicited by TLR4 and perhaps TLR2.

The main ligand of TLR4 is LPS, the major component of the outer membrane of Gram‐negative bacteria. LPS is composed of lipids and carbohydrates, with a high level of structural complexity, and consists of 3 different components: the O antigen, an O‐polysaccharide chain of variable length; the core oligosaccharide; and lipid A, which contributes to most of the immunostimulatory activity of the molecule.^39^ The O antigen is specific for each bacterial strain and affects colony morphology; microbial variants with full‐length O‐polysaccharide chains form smooth colonies, whereas those lacking or carrying reduced chains form rough colonies.^40^

**Figure 1:**
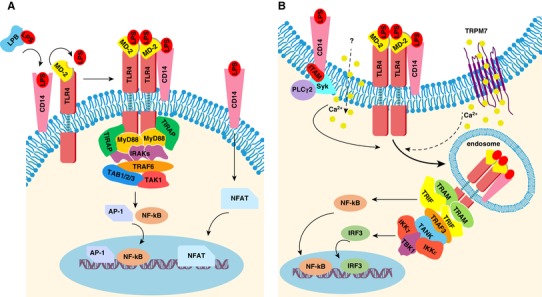
**TLR4 plasma membrane and endosome signaling**. **A**) LPB protein extracts LPS from the bacterial cell wall and transfers it to CD14. In the presence of LPS, CD14 allows the translocation of the TLR4‐MD‐2 complex to lipid rafts, where it dimerizes. Then the formation of the “myddosome” complex (containing TIRAP, MyD88, and IRAKs) occurs. IRAKs recruit TRAF6, which interacts with TAB1/2/3 and TAK1 for the activation of NF‐κB and AP‐1. CD14 binds directly to LPS and induces a signal that leads to the activation of NFAT transcription factors. **B**) The LPS receptor complex is internalized through a CD14‐dependent mechanism, involving ITAM‐bearing molecules, Syk tyrosine kinase, and PLCγ2. Calcium mobilization from the extracellular space via TRPM7 is also required, at least in part. In the endosome, TRAM‐TRIF adaptor molecules bind to TRAF3, which interacts with TANK to recruit IKKs and TBK1, which activate IRF3

Despite TLR4 being the central mediator of innate and adaptive immune responses induced by LPS, endotoxin recognition also requires other surface molecules. Indeed, TLR4 forms the LPS multi‐receptor complex with LPS binding protein (LPB), glycosylphosphatidylinositol (GPI)‐anchored protein CD14, and myeloid differentiation 2 (MD‐2).^12^ LPB is a soluble protein that binds large LPS aggregates on the bacterial cell wall,^41^ leading to LPS disaggregation and the presentation of monomers to CD14.^42^ Upon LPS stimulation, CD14 promotes re‐localization of the TLR4‐MD‐2 complex to lipid rafts, which are enriched in PIP2 (phosphatidylinositol 4,5‐bisphosphate).^43^, ^44^ At this point, TLR4 dimerizes and can initiate signal transduction from both the plasma membrane and the endosome; on the plasma membrane, PIP2 binds to TIRAP and mediates the activation of the Myd88 pathway^45^ (Fig. 1A), whereas TLR4 activates the TRAM‐TRIF pathway upon internalization into the endosome (Fig. 1B).^36^ The coordinated actions of all the proteins of the LPS multi‐receptor complex, combined with the ability of CD14 and MD‐2 to sense and bind LPS, even at picomolar concentrations, ensures the detection of bacteria with high sensitivity.^46^

Recent data have shown that not only TLR4 but also CD14 can sense LPS. Indeed, the surface molecule CD14 alone is able to activate a signaling cascade in response to LPS, leading to activation of the NFAT in DCs (Fig. 1A).^47^ Moreover, several studies have demonstrated that intracellular LPS activates the formation of a caspase‐11‐dependent noncanonic inflammasome.^48^, ^49^, ^50^ More recently, Shi et al. have identified the receptors for intracellular LPS by showing that caspase‐11 in mice and caspase‐4 and ‐5 in humans directly bind LPS.^51^

## 4 TLR4 PLASMA MEMBRANE AND ENDOSOMAL SIGNALING

TLR4 activates 2 signaling pathways. From the plasma membrane, the receptor induces the TIRAP‐MyD88 pathway, which activates NF‐κB and AP‐1. From the endosome, TLR4 initiates the TRAM‐TRIF pathway, leading to the activation of IRF3, the production of type I IFNs, and a late wave of NF‐κB activation.^36^

As recently reviewed by Brubaker et al.,^12^ upon TLR4 activation, TIRAP facilitates the interaction of MyD88 with TLR4 via its TIR domain, leading to the formation of the so‐called “myddosome,” a large molecular platform composed of MyD88, TIRAP, and IRAK proteins.^52^, ^53^ IRAK4 activates both IRAK1 and IRAK2, which, in turn, recruit TRAF6. TRAF6 interacts with TAB1, TAB2, TAB3, and TAK1, regulating the activation of NF‐κB and AP‐1 via IKKs and MAPK, respectively (Fig. 1A).^12^

After the first wave of NF‐κB and AP‐1 activation, the bipartite sorting signal of the adaptor protein TRAM controls trafficking of the entire LPS receptor complex to the endosomal compartment.^36^ During internalization, the TIRAP‐MyD88 complex is released from the invaginating plasma membrane, allowing TRAM‐TRIF to engage the TIR domain of TLR4.^36^ The first step for TRIF‐dependent IRF3 activation entails the recruitment of TRAF3 to TRIF. In turn, TRAF3, by interacting with TANK, recruits TBK1 and IKK‐ε; this complex then activates IRF3 and induces the production of type I IFNs (Fig. 1B).^54^, ^55^ It has become clear over the last 10 yr that both plasma‐membrane and endosome signaling of TLR4 are required for the full response to LPS, highlighting the importance of both the internalization process and the molecules involved. In addition to the 2 main signaling pathways of TLR4, TLR4 intracellular signaling boosts micropinocytosis and antigen presentation^56^, ^57^ and, recently, it has also been shown to be involved in the recognition and uptake of apoptotic cells.^58^

## 5 ENDOCYTOSIS OF TLR4

After the first wave of NF‐κB activation, the LPS receptor complex is internalized and redirected to the endosome. A series of studies have underlined the central role of CD14 in this process and have demonstrated that the production of type I IFN depends on CD14, highlighting the essential role of CD14 in the induction of the type I IFN‐mediated response against Gram‐negative bacteria. In particular, it has been shown that the TLR4‐CD14‐TRAM‐TRIF pathway is required for the induction of IFN‐γ production in NK cells during Gram‐negative bacterial infections.^59^ Jiang et al. demonstrated that CD14 is absolutely required for both activation of the TRAM‐TRIF pathway and the production of type I IFN in response to smooth and rough LPS, despite its being dispensable for the detection of high doses of LPS by the complex.^60^

Two studies have described how CD14 orchestrates endosomal re‐localization of the LPS complex: CD14‐dependent TLR4 endocytosis, called “inflammatory endocytosis,” is mediated by the activation of the tyrosine kinase Syk and phospholipase Cγ2, of which the activation is regulated by ITAM and the adaptors DAP12 and FcεRγ (Fig. 1B).^56^, ^61^

A recent study has proposed that the chanzyme TRPM7 (transient receptor potential cation channel, subfamily M, member 7) is involved in LPS‐induced TLR4 endocytosis in macrophages by mediating calcium influx (Fig. 1B).^59^ Indeed, the authors showed that both genetic deletion of *trpm7* and pharmacologic inhibition of the channel abolish, at least partially, the calcium influx in response to LPS, preventing TLR4 internalization.^62^ However, TRPM7 may control the recycling of TLR4 rather than its internalization.^63^ Further research is needed to clarify the mechanism by which TRPM7 regulates TLR4 endocytosis.

Recently, a study has clarified how TLR4 is selected as cargo for endocytosis.^64^ Starting from the observation that the endocytosis of CD14 occurs constitutively in resting cells, the authors hypothesized that the tail of TLR4 is dispensable for the initiation of TLR4 internalization. As a TLR4 mutant lacking intracellular domain did not abrogate the process, the authors inferred that the cargo‐selection agent resided in the extracellular portion and hypothesized the involvement of the interaction between TLR4 and MD‐2. Indeed, they discovered that both direct binding of MD‐2 to the TLR4 ectodomain and MD‐2‐dependent TLR4 dimerization promote TLR4 endocytosis.^61^ Thus, MD‐2 plays a key role in TLR4 signaling by coordinating both signal transduction and endocytosis.

Depending on the cell type, the endocytosis of TLR4 involves different players. For example, a specific role for CD11b in promoting the endocytosis of TLR4 has been found in DCs but not in macrophages, as the absence of the integrin affects the process only in DCs.^65^ Notably, CD11b is required for the correct internalization of TLR4 only in cells with low levels of CD14.^69^ Indeed, the treatment of CD11b‐deficient DCs with CpG DNA leads to higher levels of expression of CD14 that compensate the TLR4 internalization defect of the cells. However, CpG treatment does not rectify the defect that the cells have in the TRIF/IRF3 pathway, showing that CD11b plays another role in addition to the modulation of TLR4 trafficking.^65^

The endocytosis of TLR4 is negatively regulated by the metallopeptidase CD13. CD13 is up‐regulated in the presence of LPS and inhibits TRIF signaling in DCs, as shown by higher levels of TLR internalization in CD13‐deficient cells.^66^ How CD13 negatively regulates TLR4 trafficking is not yet clear, but neither the inhibition of MD‐2 nor the inhibition of CD14 seem to be involved.^66^

Perkins et al. described a new negative‐feedback loop driven by the PGE2‐EP4 axis that specifically inhibits TLR4‐mediated TRIF‐dependent type I IFN production by regulating TLR4 trafficking. Specifically, PGE2 is rapidly secreted and acts in an autocrine‐paracrine regulatory loop in response to bacterial LPS.^67^

Finally, it is worth noting that pathogenic and commensal bacteria prevent TLR4 endocytosis by producing dephosphorylated LPS to evade detection and CD14‐mediated transport to the endosome.^64^

## 6 IT IS ALL ABOUT TRAFFICKING: THE PATH OF TLR9 INTO THE ENDOLYSOSOMAL SYSTEM

The complexity of the endosomal system fine‐tunes the immune response by ensuring the correct compartmentalization of intracellular TLRs and their ligands.

**Figure 2:**
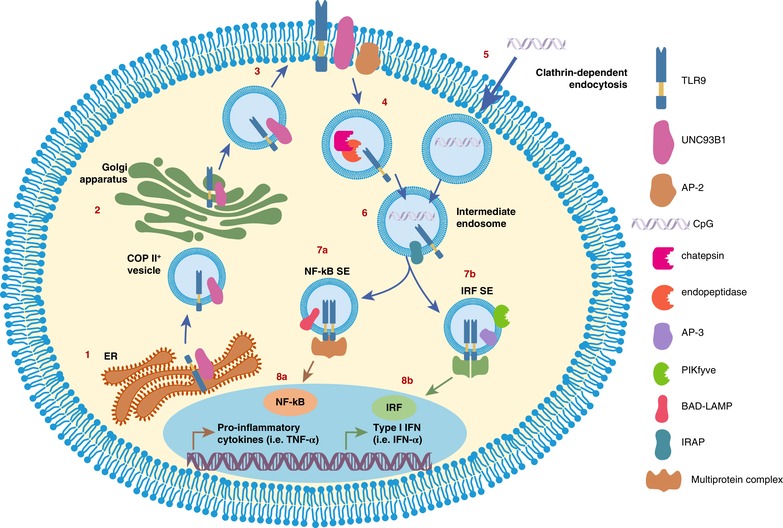
**TLR9 intracellular trafficking and pathway**. At steady state, TLR9 co‐localizes to the ER with UNC93B1 (1). TLR9 follows the secretory pathway through the Golgi (2) to reach the plasma membrane (3) via COPII+ vesicles. TLR9 is endocytosed in a clathrin‐dependent manner via AP‐2 to enter the endolysosomal system, where acidification of the endosomes allows proteolytic cleavage of TLR9 by cathepsins and endopeptidases (4). In parallel, the TLR9 ligand, CpG, is endocytosed in a clathrin‐dependent manner (5) and meets its cognate receptor (6). Here, the pathway bifurcates. IRAP^+^ early endosomes carry both TLR9 and CpG, and the presence of this aminopeptidase results in reduced immune activation, as IRAP interacts with actin‐nucleation factors to slow TLR9 trafficking to the late endosomes. In human pDCs, BAD‐LAMP facilitates the trafficking of TLR9 and CpG

In resting cells, TLR9 is localized to the ER^30^, ^68^, ^69^ and requires endosomal shuttling to initiate signal transduction. TLR9 engagement can culminate in 2 outcomes: the activation of IRF in the IRF‐signaling endosomes (IRF‐SE) and the activation of NF‐κB in the NF‐κB‐signaling endosomes (NF‐κB‐SE). Thus, the TLR9 signaling pathway has been defined as “bifurcated.”^70^ Specifically, the trafficking of TLR9 and its ligand to the IRF‐SE leads to the production of type I IFN, whereas localization to the NF‐κB‐SE induces the expression of pro‐inflammatory cytokines, via IRF and NF‐κB, respectively (see Fig. 2).

Several checkpoints control TLR9 shuttling through vesicles and involve several membrane and adaptor proteins,^70^ actin‐nucleation factors, cytoskeletal remodeling proteins,^71^ lysosome‐ or vesicle‐associated membrane proteins (LAMPs and VAMPs),^72^ and folding chaperones. For example, UNC93B1 facilitates TLR9 trafficking from the ER to the Golgi^32^ and then controls the loading of TLR9 into COP II^+^ vesicles, which deliver the receptor to the plasma membrane.^31^ At the cell membrane, UNC93B1 recruits the adaptor protein AP‐2 via its C‐terminal YxxΦ motif and mediates clathrin‐dependent internalization of TLR9, leading to localization of the receptor to early endosomal compartments.^68^, ^69^ The early endosomes that contain TLR9 and its ligand are still poorly characterized.

The brain and DC‐associated LAMP‐like molecule (BAD‐LAMP) is a member of the lysosome‐associated membrane glycoproteins and controls, together with UNC93B1, the trafficking of TLR9.^72^ It is expressed by pDCs, which produce the largest amount of type I IFNs in response to viral infections.^73^, ^74^ Indeed, in human pDCs, BAD‐LAMP co‐localizes with UNC93B1 from the ER to an endosomal hybrid compartment, the IRF‐SE, which expresses both VAMP3 and LAMP2. From the IRF‐SE, BAD‐LAMP directs and promotes the trafficking of TLR9 to a LAMP1^+^ late endosome, the NF‐κB‐SE, leading to the production of pro‐inflammatory cytokines.^72^

In murine bone marrow‐derived DCs (BMDCs), the intermediate VAMP3^+^ endosome contains insulin‐responsive aminopeptidase (IRAP), a type II transmembrane protein. IRAP is involved in antigen processing for cross‐presentation via MHC I,^75^, ^76^, ^77^ but recently a new role in TLR9 trafficking has been proposed, as TLR9 and its ligand are cargo of IRAP^+^ intermediate endosomes.^71^ In IRAP^+^ endosomes, IRAP interacts with forming‐homology‐domain‐containing protein 4 (FHOD4), a protein that promotes actin assembly on endosomes; their interaction delays TLR9 trafficking and limits the shuttling of TLR9 to LAMP^+^ lysosomes by enhancing endosome retention. Accordingly, IRAP‐deficient DCs show higher levels of IRF7 and NF‐κB activation than wild‐type DCs (see Fig. 2).^71^

### 6.1 The game changer: AP‐3

The signaling cascade triggered by TLR9 depends on intracellular trafficking of the receptor. APs select the cargo in the vesicles and, specifically, AP‐3 determines whether TLR9 is addressed to IRF‐SE to promote type I IFN production.^70^ Indeed, AP‐3 is required for the formation of lysosome‐related organelles (LROs),^78^, ^79^ in which one of the two TLR9 signaling cascades occurs, depending on the origin of the cell.

Iwasaki et al. have proposed that TLR9 from the Golgi enters NF‐κB‐SEs (characterized by the expression of VAMP3 and PI(3,5)P_2_ and the lack of LAMP2 expression), where it promotes the transcription of pro‐inflammatory cytokines in murine bone marrow‐derived pDCs. In NF‐κB‐SEs, AP‐3 interacts with TLR9 and induces shuttling of the receptor to LAMP2^+^ LROs (IRF‐SEs), resulting in the production of type I IFN.^70^

Consistent with Iwasaki's model, Blasius et al. found that AP‐3 is essential for the induction of type I INF production, specifically in pDCs.^78^ They observed that pDCs derived from mice with mutations in AP‐3b1 (*Ap3b1* ^pearl/pearl^ and *Ap3b1* ^bullet gray/bullet gray^ 80) fail to produce both type I IFNs and TNF‐α upon TLR9 activation. However, the release of cytokines in cDCs isolated from the same animals was unaffected,^78^ confirming the intrinsic difference between pDCs and cDCs. In accordance with these results, the pDCs of Hermansky‐Pudlack syndrome (HPS) type 2 patients, with AP‐3 defects, exhibit reduced IFN‐α production upon challenge with HSV‐1.^81^

Conversely, Combes et al. reported that the production of type I IFNs was unaffected by silencing of AP‐3 in a human pDC cell line (CAL‐1). However, they demonstrated that the AP‐3 complex contributes to shifting endosomal compartments by promoting TLR9 and BAD‐LAMP access to late endosomes for the activation of the NF‐κB pathway.^72^

Finally, several studies have suggested that AP‐3 is regulated by the phosphoinositide 3‐phosphate 5‐kinase (PIKfive), a kinase that controls the status of the phosphorylated derivatives of phosphatidylinositol (PI), key components of cell membranes.^82^ It has been shown that PIKfive and phosphorylated PIs regulate TLR signaling by orchestrating their intracellular pathways.^83^, ^84^ Specifically, in NF‐κB endosomes, PIKive converts PI(3)P to PI(3,5)P_2_,^85^ which recruits and interacts with AP‐3.^86^ Thus, PIKfive ensures the correct trafficking of TLR9 and CpG to type I IFNs‐SE^87^ by guaranteeing both the recruitment of AP‐3 and the generation of LROs.^88^ Moreover, an additional role of PIKfive in pDCs has been suggested, as its inhibition suppresses both IRF7 and NF‐κB pathways in pDCs, whereas it abrogates only type I IFN production in cDCs.^88^ The role of AP‐3 in generating LROs thus appears to be clear, although the signaling cascade that is triggered from the LROs is still a matter of debate.

## 7 IT IS ALL ABOUT TRAFFICKING: CPG IS LOOKING FOR A RECEPTOR

The trafficking of TLR9 to endosomal compartments is of utmost importance for the initiation of signaling cascades (see Fig. 2). However, CpG also requires controlled shuttling to endolysosomes to encounter TLR9 and activate the pathways. Indeed, upon CpG stimulation of human DCs, the DNA undergoes rapid clathrin‐dependent and caveolin‐independent internalization into vesicles that localize in juxtanuclear areas.^68^ Then, TLR9 is actively shuttled to CpG‐rich compartments because of the recruitment of MyD88 in the vesicles. Two studies have also shown that CpG trafficking affects the efficiency of TLR9 signaling, as the abrogation of CpG trafficking to the LAMP^+^ late compartment impairs TLR9 pathways.^87^, ^88^

Some of the molecules involved in the shuttling of CpG to the endolysosomal system are discussed below.

### 7.1 Granulin

CpG interacts with a co‐receptor that delivers it to the endolysosomes: granulin.^89^, ^90^ Granulin coordinates CpG trafficking to TLR9‐rich vesicles, where it promotes the interaction between the ligand and the C‐terminal domain of TLR9, guaranteeing activation of the signaling cascade.^89^

Granulin is a cysteine‐rich protein^91^ involved in several biologic processes, such as wound healing,^92^ embryonic development, and cell growth.^93^, ^94^ Park et al. first identified granulin in RAW macrophages by mass spectrometry, as it was among the polypeptides that co‐immunoprecipitated with TLR9 in protease‐inhibited RAW macrophages.^90^ This study also confirmed the importance of granulin for the activation of TLR9 signaling. The addition of granulin to the macrophage culture increased TNF‐α production only upon CpG stimulation, whereas the removal of secreted granulin reduced the amount of TNF‐α released by the cells.^90^ Moreover, BMDMs and pDCs isolated from granulin‐deficient mice exhibit impaired TNF‐α and IL‐6 production upon CpG treatment.

Several studies have also suggested that granulin plays a role in autoimmunity. Tanaka et al. found high levels of granulin in the serum of patients with SLE^95^; Xiong et al. later confirmed the same result in a mouse model of SLE and linked the increased amount of granulin to an aggravation of lupus nephritis, a clinical manifestation of SLE.^96^, ^97^ Therefore, granulin appears to worsen the autoimmune status of both humans and mice. Indeed, Xiong et al. demonstrated that granulin promotes the shifting of macrophage polarization toward an M2b phenotype, leading to increased production of pro‐inflammatory cytokines, such as TNF‐α, IL‐6, and IL‐1β.^96^ As TLR9 pathway activation in macrophages induces M1 polarizing signaling,^98^ it is likely that granulin‐mediated M2b polarization involves an additional receptor or an alternative mechanism. In addition, Chen et al. focused on macrophages, excluding pDCs and B cells from the scenario of activated lymphocyte‐derived DNA‐induced lupus nephritis.^96^ Hence, the role of granulin in autoimmunity appears to be poorly characterized, in particular regarding type I IFN production upon TLR9 engagement in pDCs. Overall, these results highlight the unclear role of granulin in both the TLR9 pathway and autoimmune diseases.

### 7.2 HMGB1

Another co‐factor that facilitates DNA sensing is high‐mobility group box 1 (HMGB1). HMGB1 is a multifunctional protein that resides in the nucleus and regulates chromatin structure,^99^, ^100^, ^101^ V(D)J recombination,^102^, ^103^ and gene transcription.^104^ Upon tissue damage, HMGB1 is secreted by necrotic cells,^105^ whereas immune cells actively release it during infections and when stimulated by inflammatory mediators.^106^

To date, only a few studies have investigated the role of HMGB1 in the immune response. Tian et al. showed that HMBG1 binds to bacterial and mammalian DNA, as well as CpG by treating peripheral blood mononuclear cells (PBMCs) with sera from SLE patients.^107^ The authors suggested that HMBG1 may catalyze the TLR9‐mediated response to DNA, as it enhances the stimulatory effect of CpG on pDCs by increasing the production of both IFN‐α and TNF.^107^ In addition, the authors demonstrated that, in pDCs, the HMGB1‐DNA complex binds to the receptor for advanced glycation end‐products (RAGE), which in turn interacts with TLR9, increasing the production of type I IFN production by pDCs via the internalization of DNA.^107^ The crucial role of the HMGB1‐RAGE axis in TLR9 regulation has also been confirmed by Tian et al., who treated PBMCs with sera collected from SLE patients and necrotic cell supernatants, showing that DNA complexes in the sera induced type I IFN production. This induction was abrogated by treating necrotic cells with inhibitors of HMGB1 or RAGE.^107^

Accordingly, Ivanov et al. demonstrated that HMGB1 binds to CpG in BMDCs and BMDMs and plays an essential role in enhancing the release of pro‐inflammatory cytokines.^108^ The authors showed that the augmented response was not due to increased internalization of HMGB1‐CpG, but to a more rapid interaction between TLR9 and CpG. Indeed, HMGB1 already co‐localizes with TLR9 in early vesicles in BMDMs prior to CpG stimulation and accelerates TLR9 redistribution to early endosomes in response to CpG‐ODN.^108^ As HMGB1 secretion increases when BMDMs and BMDCs are treated with CpG, it is likely that HMGB1 acts at 2 levels: in the extracellular space by binding to CpG and in the intracellular space by hastening TLR9 shuttling and, thus, catalyzing the TLR9 signaling cascade.^107^, ^108^

## 8 THE CONTROVERSIAL ROLE OF TLR9 PROTEOLYTIC CLEAVAGE EVENTS

An additional mechanism that limits TLR9 activation involves a multistep proteolytic cleavage that is required for MyD88 recruitment and the triggering of both signaling cascades.^109^ The cleavage of TLR9 occurs in endolysosomal compartments as an evolutionary strategy to prevent aberrant self‐recognition, such that the 150 kDa full‐length receptor on the plasma membrane, which is potentially in contact with self‐DNA, remains nonfunctional.

In the endolysosomes of macrophages, lysosomal cathepsins and endopeptidases, which function only at acidic pH, cleave the TLR9 ectodomain between LRR14 and 15 into an 80 kDa protein.^109^, ^110^, ^111^ Additional proteolytic events that involve asparagine endopeptidase occur in both myeloid and plasmacytoid DCs, showing that different cell types may activate specific proteolytic pathways.^112^, ^113^

Other studies have shown how TLR9 proteolysis fine‐tunes downstream signaling, by showing that alternative cleavage of endogenous TLR9 negatively regulates its signal transduction. Specifically, Chockalingam et al. described a novel proteolytic cleavage that results in the formation of soluble TLR9 (sTLR9), which binds to CpG DNA and hinders TLR9 transduction. The authors showed that the neutralization of endosomal pH had no effect on TLR9 formation, suggesting that the alternative cleavage may depend on cathepsin S, a protease active at both acidic and neutral pH.^114^

Similarly, another study found an N‐terminal cleavage product of TLR9 that negatively regulates its signaling; by binding to the C‐terminal fragment, the N‐terminal product accelerates the dissociation of C‐terminal homodimers and promotes its aspartic protease‐mediated degradation. This autoregulatory negative‐feedback mechanism may prevent excessive TLR9 signaling.^115^ In contrast, Onji et al. showed that the N‐terminal cleavage product of TLR9 is required for signaling.^116^ These discordant results highlight the complex regulation of TLR9 signaling controlled by its own processing and cleavage products.

Finally, Sinha et al. showed that the cleaved and mature form of TLR9 (the C‐terminal fragment TLR9^471‐1032^) is by itself unable to respond to CpG DNA when transfected into TLR9‐deficient macrophages or DCs. Moreover, its activity was not rescued either by the co‐expression of the N‐terminal fragment, which fails to restore the native glycosylation pattern of TLR9^471‐1032^, or inclusion of the cleavage site.^117^ These data suggest that TLR9^471‐1032^ is generated from full‐length TLR9 in the endosome in the presence of its ligand; if these conditions are not met, the active form is not properly glycosylated and may act as a negative regulator.^117^

## 9 DOWNSTREAM TLR9 ENGAGEMENT

Signal transduction begins once TLR9 and its ligand enter the endolysosomal system. TLR9 engagement leads to the recruitment of different players, depending on the cell type. In cDCs, macrophages, and pDCs, TLR9 activates the signaling cascade that culminates with the production of pro‐inflammatory cytokines, such as TNF‐α, IL‐6, and IL‐12. Instead, the receptor initiates the pathway that leads to type I IFN release primarily, but not exclusively, in pDCs. These 2 signaling cascades are discussed in detail below.

### 9.1 TIRAP: An adaptor only for the TLRs on the plasma membrane?

Several studies have investigated whether intracellular TLRs require sorting adaptor molecules, such as TIRAP, to signal. The sorting capacity of TIRAP relies on its amino‐terminal localization domain, which was initially believed to strictly localize TIRAP at the plasma membrane, in association with PI(4,5)P2.^84^, ^118^ However, the group of Jonathan Kagan shed new light on the role of TIRAP in TLR9 signaling.^119^ They challenged wild‐type and TIRAP‐knockout BMDMs and pDCs with either CpG or specific HSV‐1 strains that are sensed only by TLR9, as reported by Sato et al.^120^ Intriguingly, IL‐1β and IL‐6 production was impaired only in TIRAP‐knockout BMDMs stimulated with HSV‐1. Their results suggest that TIRAP plays a crucial role in sensing natural TLR9 ligands, such as HSV‐1.^119^ Conversely, Piao et al. reported less production of TNF‐α and IL‐6 after CpG stimulation of primary macrophages treated with 2R9, a peptide that binds TIRAP, inhibiting its binding to TIR domains. Thus, the role of TIRAP in macrophage activation upon TLR9 challenge may depend on the stimulus and may enhance myddosome formation.^121^

Bonham et al. also investigated how TIRAP influences the signaling of intracellular TLRs by stimulating wild‐type and TIRAP‐knockout pDCs. The authors chose pDCs as their in vitro model because these cells respond to infections exclusively via endosomal TLRs^119^ and allow investigation of the functions of TIRAP in the various endosome populations that generate the bifurcated pathway.^70^ Intriguingly, upon HSV stimulation, TIRAP knockout pDCs were unable to produce IFN‐α but not IL‐12p40.^119^ These results suggest that TIRAP is essential for the signaling that begins from late endosomal compartments.^70^ Finally, the authors confirmed that TIRAP can bind to multiple lipids^122^ and showed that its interaction with 3′ PIs and phosphatidylserine (PS) in the endosome is sufficient to promote the TLR9 signaling that leads to type I IFN production.^119^ Recently, Ve et al. proposed a sequential and cooperative model for the assembly of TIR‐signaling complexes. Their structural and kinetic data demonstrate that sequential monomer addition, rather than dimerization and trimerization, is more favorable, providing a more sensitive response.^123^ Javmen et al. also investigated the role of TIRAP in TLR9 signaling by screening a peptide library derived from TLR9 TIR. They uncovered inhibitory peptides that block TLR9 signaling in vitro and in vivo. In particular, they showed that the 9R34‐ΔN peptide can bind to both TLR9 TIR and TIRAP TIR, suggesting a common mode of TIR domain interaction in the primary receptor complex.^124^

### 9.2 The production of pro‐inflammatory cytokines

Once the ligand binds to the leucine‐rich repeats in the ectodomain of TLR9, the receptor undergoes a conformational change that allows the formation of homodimers and association of the TIR domains.^125^ Depending on the cell type and stimulus, the juxtaposed TIR domains recruit TIRAP^119^, ^120^ and the adaptor molecule MyD88, which interacts with IRAK4 through its N‐terminal death domain (DD).^126^ IRAK4 phosphorylates and activates IRAK1 and IRAK2, which then activate the E3 ubiquitin ligase TRAF6.^127^, ^128^ TRAF6 mediates the formation of K63‐linked polyubiquitin chains on NF‐κB essential modulator (NEMO or IKK‐γ) and itself.^129^ These chains create a scaffold for the recruitment of TAB2 and allow the formation of a multiprotein complex composed of TRAF6,^130^ NEMO, TAB2, TAB1, and TAK1.^131^ In parallel, the NEMO recruits IKK‐β, which is phosphorylated by TAK1.^132^, ^133^ Activation of the IKK proteins^134^ results in the phosphorylation of IκBs,^135^ which leads to their degradation and the translocation of NF‐κB to the nucleus. At the same time, TAK1 mediates the activation of the MAPK‐signaling cascade, resulting in the nuclear translocation of AP‐1.^136^ Simultaneously, IRF1 and IRF5 are directly activated by MyD88.^137^, ^138^ Finally, the activated IRFs, NF‐κB, and AP‐1 induce the expression of pro‐inflammatory cytokines (TNF‐α, IL‐6, and IL‐12).

### 9.3 The production of type I IFN

Several studies have shown that only pDCs produce type I IFN following TLR9 engagement; however, cDCs and macrophages can also release IFNs upon TLR9 challenge.^139^, ^140^ Here, we describe the signaling pathway in pDCs, the major producers of type I IFN.

### 9.4 pDCs and type I IFN production

Type I IFN production by pDCs is essential to protect the host against viral infections.^141^ Whether the signal from the IRF‐SE occurs sequentially or simultaneously to that triggered from the NF‐κB‐SE is still under discussion. Once AP‐3 interacts with TLR9 and shuttles from the NF‐κB‐SE to the LROs, the pathway forks.^70^ At this point, TIRAP acts as a sorting adaptor and is required for the formation of the myddosome, a multiprotein complex. MyD88 recruits IRAK4,^141^, ^142^ which then interacts with TRAF6, TRAF3,^143^, ^144^ and IRAK 1.^145^ Once this multiprotein complex has formed, IRF7 association with MyD88 and TRAF6 promotes IFN‐α production.^28^, ^29^, ^146^ In addition, IKK‐α enhances IFN‐α release via IRF7 phosphorylation.^147^ Hence, IRF7 disassociation from the complex and its translocation to the nucleus induces type I IFN transcription.

An additional player in the pathway is osteopontin (OPN), which contributes to the induction of IFN‐α production, specifically in pDCs, because they express intracellular OPN, as opposed to cDCs, which do not. Although the precise mechanism by which OPN supports type I IFN release is still unknown, it is considered to be a functional member of the multiprotein complex. Indeed, upon TLR9 engagement, it localizes near TLR9 and MyD88, favoring the IRF7 pathway.^148^ The importance of OPN in TLR9 signaling has also been confirmed by the fact that OPN‐deficient animals produce reduced levels of IFN‐α when challenged with inactivated HSV.^148^

Another pathway that supports type I IFN release is phosphoinositol 3‐OH kinase (PI3K)‐mTOR signaling. The pharmacologic inhibition of the kinase or mTOR reduces the interaction between TLR9 and MyD88 and impairs the production of type I IFN.^149^ The mechanism by which PI3K promotes IRF7 activation and translocation into the nucleus in human pDCs has not yet been fully dissected,^143^ but it is likely that PI3K acts together with other regulatory elements of the pathway.

Finally, type I IFN is a positive regulator of its own pathway; it enhances the expression of TLR9 and MyD88, further increasing its production.^72^

## 10 AUTOIMMUNITY: THE CASE OF TLR9

The etiopathogenesis of most autoimmune diseases is still unclear, as several factors may contribute to their onset, such as the presence of autoantibodies, high serum levels of type I IFNs,^150^ or increased cell death, which trigger diseases such as RA and system lupus erythematosus (SLE).^145^ As the insufficient clearance of necrotic cells in RA and SLE results in the accumulation of nucleic‐acid containing material,^151^ researchers have investigated whether TLR9 is involved in these autoimmune responses. Indeed, in contrast to TLR4, the dysregulation of TLR9 signaling has been associated with autoimmunity, even though its precise role is still a subject of debate. A study has shown that the receptors for the Fc region of IgG (FcγR) sense immune complexes and induce their entry into the endosomal system, where self‐DNA encounters TLR9. This leads to higher production of both pro‐inflammatory cytokines and type I IFNs.^152^ Accordingly, the activation of the TLR9 pathway in pDCs and autoreactive B cells has been associated with SLE and RA.^4^, ^153^, ^154^ Below, we provide a brief overview of the findings that have suggested how TLR9 may be involved in specific autoimmune diseases

### 10.1 Systemic lupus erythematosus

Several lines of evidence support the involvement of TLR9 in the onset of SLE. First, SLE patients exhibit an altered balance in the circulating subtypes of DCs, as their pDC compartment, specialized in the production of type I IFN, is more prominant than normal.^155^ Second, B cells and monocytes from SLE patients are characterized by increased levels of TLR9 expression, which correlate with higher levels of autoantibodies against dsDNA.^154^, ^156^ Moreover, aside from the crucial role of TLR7 in the pathogenesis of SLE, it appears that only TLR9 is required for one of the hallmarks of SLE: the production of anti‐DNA antibodies.^157^

Despite these results, other studies support the hypothesis that TLR9 has a protective role in the pathogenesis of SLE. For example, TLR9‐deficient mice exhibit clear lupus‐like clinical manifestations^158^ and TLR9^−/−^ autoimmune‐prone MRL/lpr animals have a shorter lifespan due to severe SLE and glomerulonephritis.^4^ Consistent with these results, another study has proposed that TLR9 acts as a negative regulator of TLR7, the main culprit of SLE pathogenesis,^158^ by competing for UNC93B1 in the ER.^159^, ^160^ Indeed, a point mutation in UNC93B1 (D34A) facilitates the association with TLR7 and dampens TLR9 activity, leading to severe systemic inflammation in Unc93b1^D34A/D34A^ mice.^160^

### 10.2 Rheumatoid arthritis

The role of TLR9 in RA is still a subject of debate. On the one hand, some studies have proposed that TLR9 worsens the severity of RA.^161^ For example, Asagiri et al. showed that the treatment of adjuvant‐induced arthritic rats with an inhibitor of cathepsin K led to defective TLR9 signaling and improvement of their pathologic state, even though the role of this protease in the TLR9 signaling pathway is still poorly understood.^153^ On the other hand, Miles et al. reported that the administration of apoptotic cells in a murine model of collagen‐induced arthritis led to a TLR9‐dependent anti‐inflammatory effect, supporting the hypothesis that TLR9 signaling is protective against RA.^162^

### 10.3 Psoriasis

Another molecule that promotes aberrant activation of TLR9, thus inducing autoimmune diseases, is the anti‐microbial cathelicidin LL37, a hallmark of psoriasis also found in synovial membranes of arthritis patients.^163^ Human LL37 is a carboxy‐terminal peptide fragment derived from the cathelicidin precursor (human cationic antibacterial protein of 18 kDa orhCAP18) and has many anti‐microbial properties.^164^ Upon tissue damage, LL37 binds covalently to self‐DNA in pDCs and facilitates DNA internalization into the endolysosomal system; once in the endosome, TLR9 may bind to self‐DNA, inducing type I IFN production and triggering the onset of psoriasis.^165^, ^166^ Also, LL37 in keratinocytes contributes to the exacerbation of psoriasis via the activation of TLR9 and the production of type I IFN.^167^, ^168^

### 10.4 Intracellular TLRs and other autoimmune diseases

Aside from the aberrant sensing of self‐DNA by TLR9, autoimmune diseases may also result from the misregulation of intracellular TLR9 trafficking. Indeed, it has recently been demonstrated that improper trafficking may be related to autoimmunity via perturbation of intracellular TLR pathways.^169^ For example, mice lacking one of the components of the SWC complex (a protein complex involved in autophagy and endocytosis and composed of Smith‐Magenis syndrome chromosome region candidate 8 SMCR8, WD repeat domain 41 WDR41, and C9ORF72) exhibit impaired intracellular TLR signaling that leads to autoimmunity reactions and systemic inflammation.^170^ Indeed, SMCR8 negatively regulates endosomal TLR signaling, and the entire complex contributes to the vesicle acidification required to degrade TLR ligands and avoid persistent stimulation.^169^ A study has also suggested that amyotrophic lateral sclerosis (ALS) and frontotemporal dementia (FTD) in humans could be caused by *C9ORF72* repeat expansion because it generates a loss‐of‐function SWC complex.^171^ Thus, the role of TLR9 in distinct autoimmune disorders is still unclear and further insights are required to shed light on the context‐dependent effects of the engagement of this receptor.

## 11 CONCLUDING REMARKS

Over the last few years, a more comprehensive picture of the plasma membrane and intracellular signaling cascades, networks, transcriptional regulation, and other processes associated with the TLR response has emerged. In this review, we have discussed up‐to‐date knowledge of the regulation of the pathways elicited by TLR4 and TLR9 and their roles in host defense and autoimmunity.

Endocytosis and protein trafficking in TLR4 signaling are recently identified regulatory mechanisms of innate immunity and many studies have focused on the identification of the molecules involved in their modulation, leading to the discovery of new players and functions. For example, CD14 and MD‐2 are now considered to comprise a novel category of regulators of innate immunity, called transporter associated with the execution of inflammation (TAXI), rather than “classic” chaperone proteins.^172^

The trafficking of TLR9 has also emerged as a crucial checkpoint of its pathway, as adaptor proteins, LAMPs, cytoskeleton stabilizers, and PI kinases contribute to guiding TLR9 signal transduction. However, some of the mechanisms behind TLR9 trafficking are still poorly understood.

Further studies are needed to fully understand the regulation of TLR9 and TLR4 signaling. An in‐depth understanding of the regulatory mechanisms would allow, for example, steering the TLR9 pathway toward a specific immune response. Moreover, TAXI and trafficking regulators may become novel targets to prevent overt inflammation and potentiate vaccines and cancer therapies.

## DISCLOSURES

The authors declare no conflicts of interest.
